# Promotion of Street-Dance Training on the Executive Function in Preschool Children

**DOI:** 10.3389/fpsyg.2020.585598

**Published:** 2020-10-22

**Authors:** Yue Shen, Qing Zhao, Yue Huang, Ge Liu, Lele Fang

**Affiliations:** ^1^School of Psychology, Liaoning Normal University, Dalian, China; ^2^The Forth Kindergarten of Shahekou, Dalian, China

**Keywords:** street-dance, training, executive function, preschool children, intervention program

## Abstract

Executive function is the center of cognitive function, emotional function, and social function, and plays an important role in children’s cognitive development. Previous studies used music, sports, and other training methods to promote the development of children’s executive function. but researchers are still exploring more comprehensive and effective training methods. Street-dance, as a comprehensive dance form integrating the characteristics of movement, music, rhythm, and so on, needs the coordination of individual sensory systems and a sense of musical rhythm and action. These are the same activity elements found in previous studies that can improve the individual executive function of children. In order to investigate the promoting effect of street-dance training on children’s executive function, this study designed a street-dance training program integrating the characteristics of each component of executive function. Sixty preschool children around the age of four (*M* = 52.4, *SD* = 3.95) participated using the pretest-posttest experimental design. The dancing group conducted street-dance training 3 times a week, 40–50 min each time for a total of 24 times; the control group did not train. We discovered that 8 weeks of street-dance training can promote the development of executive function in preschool children, and we discussed about the potential mechanism of the street dance training effects and the implications of intervention programs.

## Executive Function

Executive function (EF) is a general control mechanism that coordinates various cognitive processes while completing complex cognitive tasks to ensure that cognitive systems achieve specific goals in a flexible and optimized manner. The essence of EF is to control and regulate other cognitive processes, and its fundamental purpose is to produce coordinated and purposeful behavior (Diamond, 2013). The prefrontal cortex is the main nerve basis of EF. Its maturation process is relatively long, and it does not fully mature until early adolescence; therefore, the development of EF will continue until adulthood (Maurits et al., 2000; Segalowitz and Davies, 2004; Conklin et al., 2007).

EF provide the necessary foundation for the development of children’s learning ability and sociality. It helps children to learn and process new information, which is related to the development of children’s multiple abilities and closely related to language, mathematics, and social abilities in the field of school readiness (Zelazo et al., 2017; Micalizzi et al., 2019). Good EF helps children to meet the requirements of the family (such as obeying rules) and classroom (such as sitting in silence) (Carlson et al., 2013; Muller and Kerns, 2015). EF can predict achievement, health, wealth, and quality of life in a lifetime, which is more predictive than IQ or socioeconomic status (Moffitt et al., 2011; Moffitt, 2012). EF measured in early childhood can predict the academic performance of children after entering school (Best et al., 2011), and predict the characteristics of cognitive processing, socio-economic status, and health status of individuals after many years (Mischel et al., 1989; Casey et al., 2011; Moffitt et al., 2011). There is a large amount of evidence that it is beneficial to a successful job and career development (Leslie, 1995; Bailey, 2007), making friends and maintaining friendship (Hughes and Dunn, 1998), marriage harmony (Eakin et al., 2004), weight control (Crescioni et al., 2011), release from prison (Moffitt et al., 2011), and resistance to drug abuse (Miller et al., 2011).

Previous studies on the content structure of EF of preschool children are not completely consistent, and there are generally two views as follows: First, the one-factor model considers that the components of EF are a unified whole (Wiebe et al., 2011; Miller et al., 2012). Posner and Rothbart et al. proposed that at the age of 2–6, the various components of EF are a unified whole under the central attention system (Miller and Best, 2010). Second, some researchers believe that the components of EF are independent of each other, including two-factor models (Monette et al., 2015) and three-factor model (Espy et al., 2004; Miller and Best, 2010; Hughes, 2011; Skogan et al., 2015). According to previous researchs on the EF training of preschool children (e.g., Lakes and Hoyt, 2004; Schonert-Reichl et al., 2015; Rosas et al., 2019; Shen et al., 2019), researchers mainly focus on three basic components.

Inhibition control refers to the ability of an individual to resist temptation rather than impulse or premature action. It can help individuals choose or change their behavior independently, and get rid of the influence of impulse or habit (Diamond, 2013). IC allows people to consciously abide by social norms and avoid social disrespect, which plays an important role in forming a good and harmonious society (Diamond and Ling, 2016). Diamond (2013) proposed that IC includes three sub-components: executive attention, cognitive inhibition, and response Inhibition (Diamond, 2013). Working memory enables individuals to keep information in mind, and process and use it in their minds, which is essential for reasoning and problem-solving (Diamond and Ling, 2016). For example, in daily life, people need WM to reorder the information in the brain or to explore the relationship between information (Smith and Jonides, 1999). WM enables people to remember their own problems or evaluations when they are discussing (Postle and D’Esposito, 2015). Cognitive flexibility refers to the ability to flexibly adapt to changing needs or priorities, and view the same thing in different ways or from different perspectives (Allport and Wylie, 2000; Monsell, 2003; Van der Elst et al., 2011). If a problem-solving method does not work, CF is needed to help people “jump out of the thinking framework,” that is, find other ways to conceive or solve problems. CF is necessary to meet new and unexpected challenges and seize opportunities in case of accidents.

The age of 3–6 years is a critical period for the development of many individual psychological abilities (Deloache et al., 2010). Lenroot and Giedd (2006) proposed that 3–6 years of age is a critical period for children’s cognitive development, and also the peak period of individual brain development. As the prefrontal cortex matures, EF develops rapidly in early childhood (Gathercole et al., 2004; McCormack and Atance, 2011; Weinstein et al., 2012).

The age of 4 is an important period for the development of EF, during which the EF of individuals will undergo qualitative changes (Carlson et al., 2002; Hala et al., 2003; Zelazo et al., 2003b; Willoughby et al., 2012). Miller and Best (2010) pointed out that the first “leap-forward” development of IC ability was between 3 and 4 years old, and advanced cognitive inhibition such as emotional control and switching developed after 4 years old. The first “leap-forward” development of WM was between the ages of 4 and 6, and 4 was a key turning point in the development of WM (Hughes, 1998). CF is developed on the basis of WM and IC. Children begin to show CF until about 4 years old (Zelazo et al., 2003b). Therefore, 4 years old is the most critical turning point in the development of children’s EF.

A large amount of evidence shows that EF is plastic (Diamond et al., 2007; Diamond and Lee, 2011). It can be improved through preschool intervention. The efforts made to improve EF before entering school have a great impact on the growth of children’s EF after entering school (Sasser et al., 2017).

Due to its importance to individual development, in recent years, many researchers have studied the intervention programs to improve children’s EF. In general, it can be divided into music training and sports training.

### Music Training

Music training is a kind of intensive activity involving multi-sensory organs, involving auditory, visual, somatosensory, motor system, attention, memory, executive function, and other related cognitive systems (Geiser et al., 2009). Some studies have shown that music training can stimulate the activity of the cerebral cortex, and short-term training for 6 months can significantly improve behavior and change the growth of synapses, showing that music training can change the plasticity of the brain (Sylvain et al., 2009). Shen et al. (2019) conducted 12 weeks of integrated music training for young children who had not previously received any music learning, the control group did not train. After the training, children in the music training group and children in the control group were tracked and tested. The results show that integrated music training can promote the IC, WM, and CF of children. Jaschke et al. (2018) selected 147 students (average age is 6 years) from 6 primary schools in the Netherlands to conduct longitudinal music training intervention and measured the subjects’ academic performance and EF at five-time points. Research shows that longitudinal music training can promote academic achievement, and EF plays an intermediary role between music training and academic performance.

### Sports Training

EF development is a multi-stage process. From the beginning of sensory-motor interaction, these behaviors gradually form basic cognitive ability and then, form EF (Wu et al., 2017). Sports training can be game training, aerobic training, martial arts training, and so on. Rosas et al. (2019) specially designed 32 games to promote the development of EF in which 35 children were trained for 3 months. The results showed that compared with the control group, the EF of children in the training group was significantly improved. A large number of studies have shown that aerobic exercise can effectively improve prefrontal cortex function and EF (Hillman et al., 2008). Hillman et al. (2009) conducted a study on the influence of children’s short-term exercise on their cognitive control and learning performance. The results showed that one-off moderate-intensity aerobic exercise can promote the improvement of children’s attention executive control, and has a special role in the support system of cognitive health (Hillman et al., 2009). Compared with single aerobic exercise, martial arts training can involve both sports and character development, which can improve EF more effectively. Lakes and Hoyt randomly assigned children aged 5–11 to the Taekwondo training group and general physical education group. It was found that compared with children in the general physical education group, children in the Taekwondo group had significantly improved WM and IC (Lakes and Hoyt, 2004).

Researchers also combine music and movement for training, such as dance training and rhythm training. Puckering et al. (2014) provided 3 weeks of music or dance training to 50 children from 6 to 9 years of age, while 24 children in the control group did not receive any training. The results showed that the task conversion ability of the children in the training group was improved. Rhythm teaching has an enlightening role in dance education, and to lay a good foundation for dance, the rhythm training is generally carried out first. Research has shown that rhythmic skills are related to cognitive abilities such as WM (Tierney and Kraus, 2013; Woodruff Carr et al., 2014), so rhythm training may promote broader cognitive abilities (Schwartze and Kotz, 2013; Gordon et al., 2015; Kotz and Gunter, 2015).

In recent years, researchers have explored more and more training methods to promote children’s EF. Diamond and Ling summarized previous studies on children’s EF training and came up with several principles for effective promotion of EF (Diamond and Ling, 2016), for example: (1) Although training may seem highly transference, it is often closely related to trained cognitive function. Therefore, to avoid the predictability of test tasks, it is recommended to use various tasks that require multiple cognitive skills. (2) The length of training time is important; with more training weeks and higher training frequency, the course effect becomes better. (3) EF needs to be constantly challenged. The training process needs to gradually increase the difficulty to see the improvement of EF. (4) Individuals with poor EF often see more improvement in training. (5) It is necessary to analyze as many intervention factors as possible to determine whether the results obtained are due to the training itself or other factors related to the training. In short, by analyzing the intervention programs that successfully promote the development of EF, we found that no matter which training method is based on, we need to refer to these successful promotion principles.

In conclusion, previous researchers have found that the internal causes of promoting EF may be sensory integration, neural inhibition, executive attention, memory, etc. The more comprehensive the factors are, the better the training effect is. Therefore, researchers are still trying to explore various possible comprehensive training methods, and the design scheme is also increasingly inclined to include a variety of form features. Especially in the special group of children aged 3–6, the attractiveness and participation of the design scheme will also affect the training effect. Therefore, this study will take street-dance, which integrates the characteristics of movement, music and rhythm, as a carrier to promote the development of children’s EF.

## Effect of Street-Dance Training on Children’s Executive Function

Street-dance is an umbrella term comprising different dance styles and techniques in American hip hop culture at the end of the 1960s (Macmillan Dictionary, 2007). In a narrow sense, street-dance is a kind of sports activity. It is a combination of a series of intensive activities and low-intensity sports. The characteristics of this dance include rapid changes in footsteps, strong explosive power and a combination of strong rhythmic music (Gustavo et al., 2011), so it can better improve the coordination ability of practitioners, and fully exercise the small joints and small muscle groups that the body does not often move to. Any kind of street-dance needs to show each movement in the correct order, appropriate duration, and appropriate time and space. This process requires the participation of sensory, motor systems, and integrated brain regions, and involves advanced cognitive processes.

Street-dance can improve motor control, attention, cognition, and physical fitness, and has an impact on human physiology and psychology.

### Impact on Human Physiological Development

Children can improve their athletic ability by doing street-dance exercise. They can develop physical qualities such as agility, speed, strength, and coordination (Grčić et al., 2014). In addition, street-dance helps to improve the flexibility of the nervous system. Through fast-paced changes and shocks, it can train children’s ability to respond and the brain’s comprehensive ability to analyze limb movements.

### Impact on Human Psychological Development

When learning street dance, children not only need to listen to music and memorize the rhythm, melody, emotion, etc. of dance music, but also need to understand the street dance movements, listen carefully to the explanation of the movement essentials, pay attention to observation, and remember the action requirements and movement trends. These contents train children’s advanced cognitive processes such as vision, hearing, movement, and memory. In the street dance practice, it is necessary for practitioners to overcome their own habitual behaviors and maintain a high degree of cognitive conversion and memory, which are important parts of children’s EF.

Dance training is a comprehensive training that combines music, aerobic exercises, and rhythm exercises. It involves the practice of many skills, such as coordination with music, retrieval of movement sequences, enhancement of spatial perception and memory, and EF (Kosmat and Vranic, 2017). Studies have shown that dancing is more beneficial than repeated physical exercises, thereby activating the plasticity mechanism of the brain to a greater extent (Dafna et al., 2016). Although the research on street-dance training is lacking, some of the research on high-frequency rhythmic dances, which are similar to street-dance performances, found that dance is conducive to the development of the individual EF. For example, Lakes et al. (2019) conducted ballet dance training for children with cerebral palsy for 5 weeks, and found that it can effectively improve children’s IC ability. Carmen et al. (2020) found that Salsa dancers performed significantly better than the control group in EF-related tasks. Aguiñaga et al. (2018) found that through 16 weeks of Latin dance training, people’s physical activity level, fitness level, and EF can be improved. In addition, Latin dance training is also conducive to improving the overall cognitive ability of individuals (including EF, situational memory, WM) (Marquez et al., 2017). Zumba is a kind of sports project based on aerobic exercise, which integrates Cha Cha dance, Salsa dance, and other South American dance forms. It is found that after Zumba intervention, individual WM and motor function have been significantly improved (Norouzi et al., 2019).

In the past, there were many methods to promote children’s EF, but some training methods also had some weaknesses. For example, some research results of music training can prove that music training can promote the development of EF, but the content of music training is more comprehensive, including instrument training, learning of music rules, etc. it is not clear what content promotes the development of EF. Although aerobic or physical training can see the impact on children’s behavior, it may be boring and make children’s interest decline. The dance form integrating music and body is conducive to integrating advantages, and the training effect can be judged by behavior according to the change of music and the coherence of dance movements. As ballet, Cha Cha and Latin dance require children to have a higher dance foundation, and they are more difficult to learn. Zumba’s exercise intensity is relatively high, and it is not suitable for training in early childhood. Compared with other dance forms, street-dance training has a wider target audience; its acceptance and attractiveness are more prominent, which are coupled with low-cost benefits, no venue restrictions, and other advantages. It is convenient to carry out. So this research explores the impact of street dance on the promotion of children’s EF, which is also the innovation of this article.

In summary, this research refers to some principles that have successfully promoted EF, designed a set of street-dance courses suitable for children, and conducted 8-week street-dance training for children. We used the pretest-posttest experimental design of the street-dance training group and the control group. We also used the Go-nogo task to measure the cognitive control of children, the attention network test (ANT) to measure the executive attention of children, the dimensional change card sort (DCCS) task to measure the cognitive flexibility of children, and backward digit span task to measure the WM of children. The street-dance training group conducted training 3 times a week, 50 min each time for a total of 24 times. The control group did not perform any training and normally participated in the class activities. We have hypothesized that street-dance training can promote the development of young children’s EF.

## Method

### Participants

In order to control the ecological validity and the homogeneity of variables (the kindergarten living environment, daily schedule, daily activities in the kindergarten, etc.), we randomly selected two classes in the same-age class of a medium-level public kindergarten in Dalian. 30 children in one class as the street-dance training group (M = 52.4 months, SD = 3.95 months, 15 boys and 15 girls), and 30 children in the other class as the control group (M = 55.68 months, SD = 4.23 months, 15 boys and 15 girls). There was no significant difference in the age of the street-dance training group and the control group (p > 0.05). All participants were healthy and developing normally. Parents have no background in dance-related occupations, and all parents of participating children have given informed consent.

### Experimental Design

A 2 × 2 experimental group and a control group were used for the pretest-posttest design. Among them, the group (street-dance training group/control group) is the inter-subject variable, and the test type (pre-test/post-test) is the intra-subject variable. The street-dance training group and the control group have not participated in similar training before. The interval between pre-test and post-test is about 2 months, and the same test task is used for pre-test and post-test. We choose the time of street-dance training is the time period that the kindergarten stipulates for children to carry out free activities every day. Therefore, while the street-dance training group participated in the street-dance training 3 times a week, 40–50 min each time for a total of 24 times, the control group carried out normal random free activities according to the requirements of the kindergarten.

### Training Program

#### Design of Street-Dance Training

We referred to Diamond’s requirements and principles of successfully promoting EF. Then, according to the characteristics of three sub-components of children’s EF (WM, CF, IC), combined with the three ways of sports affecting EF (movement characteristics, movement scenarios, movement intensity), merged the characteristics of music training and sports training, and designed a set of street-dance training intervention courses suitable for the cognitive development characteristics of 3–6 years old children.

The movement characteristics of street-dance training are arranged around three sub-components of EF: (1) when reflecting the WM factor, we should pay attention to selecting the actions that need to be learned in the new task from the actions that have been learned. (2) When reflecting on the CF factor, we should emphasize that the design of the movement should include multiple tasks to train children to flexibly switch between different tasks. (3) When reflecting on the IC factor, it is important to emphasize the removal of habitual actions and dominant actions.

The movement scenarios of street-dance training are also designed around three sub-component functions of EF. The scenarios are created by the teacher’s password and organizational form: (1) When developing the WM function, teachers’ password and actions require children to extract the actions needed by the current task from memory, so that children can change from passive acceptance to active acquisition. (2) When developing the CF function, in the face of multi-tasking movements accompanied by music, children should extract corresponding movements according to different time points to show a whole set of street-dance actions coherently. (3) The formation change is designed to make the children break the habitual actions and develop the IC function.

The movement intensity of street-dance dance training adopts moderate-intensity training, lasting for 50 min (10 min to organize), and the exercise frequency is 3 times a week. The physiological performance of children reaches a little shortness of breath, and the body slightly perspires. Studies have shown that moderate-intensity aerobic exercise can improve EF since this exercise can also improve inhibition function and conversion function (Hillman et al., 2009).

The training teacher of this street-dance training is a female teacher with 3 years of practical experience in street-dance teaching and psychological knowledge background. She has a relatively solid theoretical basis of street-dance teaching and practical experience in children’s street-dance teaching. She could better implement the guiding ideology of the street-dance training activity program and mobilize the enthusiasm of children to participate in street-dance training. The training place is an activity classroom in kindergarten.

### Specific Content of Street-Dance Training

The training content selected in our study is based on the *Chinese Street Dance Art Education Examination* textbook and the characteristics of the three sub-components of EF; it is modified by professional street-dance teachers and psychology teachers.

The purpose of the training content is clear. The training movements are all within the scope of children’s receptive ability. The training content is mainly based on large-scale movements, supplemented by small joint movements. The difficulty of the action rules is gradually increased, and the movement is arranged based on the rhythm of 2/4 beat, 3/4 beat, and 4/4 beat. The music melody combined with street-dance training is active and lively. The whole program focuses on the regularity, flexibility, integration, and interest of street-dance training.

Through the content of street-dance training courses, children’s interest in street-dance is increased; children are encouraged to take the initiative and actively participate so that they can experience pleasure in participating in street-dance training, and then, be able to observe training rules, restrain impulsive behavior, recognize and memorize street-dance movements, and flexibly use learned movements.

The 8-week hip-hop dance training content is the first level dance in the Chinese Dance Association Street Dance Art Examination. We selected two dances, and the dance movements ranged from easy to difficult, through continuous challenges to achieve the training of children’s EF. Specifically, it is divided into 2 stages: first, 1–4 weeks to learn the simple dance “*Hip-hop youth*” and then, 5–8 weeks to learn the more complex dance “*Machine dance*.” And the action teaching of each dance is a gradual process of “action—action combination—music combination.” Specific street-dance training contents are shown in Tables 1 and 2.

**TABLE 1:** Training content of simple dance “*Hip-hop youth*”.

Weeks	Beat	Training content
1	1	Neck isolation, chest isolation
	2	Leg isolation(1)
	3	Leg isolation(1), chest isolation, crotch isolation
2	4	Shoulder isolation
	5	Formation transformation
	6	Chest isolation, heel and toe isolation
3	7	Heel and toe isolation, chest isolation
	8	The reverse movement of the seventh beat
	9	Shoulder isolation combination with footsteps
4	10	Leg isolation(1), heel and toe isolation
	11	Weight shift
	12	Leg isolation(2)

**TABLE 2:** Training content of complex dance “*Machine dance*”.

Weeks	Beat	Training content
5	1	Arm control
	2	Arm control, chest updown
	3	Leg isolation(1)with the arm
	4	Leg isolation(1), heel and toe isolation, speed conversion
6	5	Crotch isolation, weight shift
	6	Chest isolation, shoulder isolation, head isolation, arm control
	7	Heel and toe isolation
	8	The reverse movement of the seventh beat
7	9	Arm control, heel and toe isolation, speed conversion
	10	Crotch isolation, heel and toe isolation
8		Review the whole dance and change the formation

### Measures

The Go-nogo task was used to measure the response inhibition component, and ANT was used to measure the executive attention component. The DCCS task was used to measure the CF component. The backward digit span task was used to measure the WM component. Specific experimental operations are as follows:

#### Go-Nogo Task

Previous researchers used the Go-nogo task to measure children’s IC level (Rosey et al., 2010; Chang et al., 2014; Sinzig et al., 2014). To stimulate children’s interest in the operation, this study self-designed a Go-nogo task. Children were shown pictures of white-eye mice or red-eye mice. To prevent systematic errors, we made a balance between go stimulation and nogo stimulation among the participants. Half of them took the red-eye mice as the go condition, and the other half responded to the red-eye mice as the nogo condition. There are 10 trials (50% for go and 50% for nogo) in the exercise experiment. After the exercise, it was ensured that the children fully understood the task content. The formal experiment was composed of 2 blocks, each block contains 80 trials (75% for go and 25% for nogo). The number of children’s correct responses to nogo condition is the response inhibition score.

#### Attention Network Test

We used an ANT compiled by Rueda et al. (2004) to measure the executive attention of children. The experimental stimulus was a row of fish that had a total of 5 fish. The swimming direction of fish was divided into the left and right sides. There were two kinds of stimuli, one was a consistent stimulus (all fish swam in the same direction), and the other was an inconsistent stimulus (the middle fish was swimming in the opposite direction to the fish on the sides). Before the experiment began, the subjects took the illustration book to explain the experiment to the children, telling the children only need to judge the direction of the middle fish. If they swam to the left, the left mouse button was to be pressed; if they were on the right side, the right mouse button was to be pressed. During the formal experiment, there was corresponding correct or wrong feedback to the child’s response. The participants completed 2 blocks, and each block contained 24 trials. A total of 48 trials, of which both consistent and inconsistent stimuli are 24 trials. The correct number of children’s responses to inconsistent stimuli is the executive attention score.

#### Dimension Change Card Sort Task

We measured the CF with the children’s standard version of the DCCS task compiled by Zelazo (2006). In this task, the subjects presented different dimensions of cards to children, including shape and color. First, children are asked to classify the cards 6 times according to the “shape” dimension, and then the cards are classified 6 times according to the “color” dimension. The above requirements were repeated three times, with a total of 36 trials (Zelazo et al., 2003a; Zelazo, 2006). There are 36 trials in total. The correct number of children’s reactions were recorded.

#### Backward Digit Span Task

We use the backward digit span task (Wechsler, 2008; Liu-Ambrose et al., 2010) to measure the WM of children. The experimental materials are numbers 0–9. The experiment is divided into two stages. In the practice stage, the subject says the numbers “1, 2” and tells the children to say the numbers “2, 1” backward. In the formal experiment, the subject randomly selects 2 numbers from 0 to 9 numbers and then, asks the child to speak the numbers backward. If the child fails, 1 point is scored. If the child succeeds, 2 points are scored. If the child succeeds, the subject continues to say 3 numbers and then, asks the child to say the numbers backward. If the child succeeds, 3 points are scored. The subject can say up to 4 numbers to the children. The total score is 9 points. The final score of children is the WM score.

### Procedures

#### Pre-test Stage

All the children in the street-dance training group and the control group received four tasks of EF, and the test lasted 2 weeks. Children participated in the experiment voluntarily and had the right to withdraw from the experiment. Before the test, the informed consent forms were distributed to kindergarten teachers and parents, and the test was conducted after receiving their consent. Before the experiment began, the subjects explained the experimental requirements to the children to ensure that the children understood the experimental tasks.

#### Intervention Stage

After the pre-test, the street-dance training group conducted an 8-week street-dance training 3 times a week, each time lasting 50 min (10 min to organize, 40 min for training). Each time, the same trainer and street-dance assistant were used for training. Trainer and street-dance assistants only conduct intervention training. During the training process, the trainer guided children to participate actively in the curriculum with an encouraging, open, and accepting attitude. Moreover, each training class had a kindergarten teacher present to maintain the discipline for ensuring the normal development of training; the control group conducted normal school activities.

#### Post-test Stage

After the intervention, all children in the street-dance training group and the control group received the same 4 EF tasks as the pre-test. The subjects of the post-test were consistent with the pre-test.

## Results

In our experiment, the scores of the 4 tasks were the dependent variables, and the time points (pre-test vs. post-test) and the groups (the experimental group vs. the control group) were independent variables. The experimental design was a 2 (time points: pre-test vs. post-test) × 2 (groups: the experimental group vs. the control group) two-way repeated-measures analysis of variance (ANOVA), in which the time points were the intra-group variables and the groups were the inter-group variables. The ANOVA mainly examined the interaction between time points and groups. In the control of unrelated variables, we took the following measures: (1) In the pretest, two classes of children were investigated to ensure that there were no extra dance training activities outside the kindergarten. (2) A homogeneity test was conducted on the pre-test data to ensure the homogeneity of children’s self-control development in the experimental class and the control class. (3) The street-dance training group carried out street-dance interventions and the children in the control group carried out free play activities at the same time, and other kindergarten activities remained consistent. The teachers did not impose additional activities on the children as a uniform requirement.

The EF task scores before and after street-dance training are shown in Table 3.

**TABLE 3:** The executive function task data of the experimental group and the control group.

	Street-dance training group		Control group	
	(*n* = 30)		(*n* = 30)	
		
	Pre-test (*M SD*)	Post-test (*M SD*)	Pre-test (*M SD*)	Post-test (*M SD*)
Go-nogo	30.97 (6.365)	34.53 (3.203)	31.8 (4.809)	30.57 (5.211)
ANT	15.07 (7.182)	20.07 (3.676)	15.77 (5.817)	16.03 (6.049)
DCCS	28.80 (5.821)	33.33 (4.444)	28.2 (6.15)	28.93 (5.037)
Backward digit span task	1.77 (1.357)	3.73 (3.028)	1.57 (1.223)	2.07 (1.874)

To better control the experimental variables, we performed statistics on the pre-test results. The results showed that there was no significant difference in the scores of the 4 tasks (Go-nogo Task, DCCS, Backward Digit Span Task, ANT) between the experimental group and the control group [*t*(58) = −0.572, 0.388, 0.6, −0.415, *p*s > 0.05]; thus, indicating that the two groups of children were homogeneous in levels of EF.

To further investigate the influence of street-dance training on children’s response inhibition, CF, WM, and executive attention, we conducted 2 (time points: pre-test vs. post-test) × 2 (groups: the experimental group vs. the control group) two-way repeated-measures analysis of variance (ANOVA), the results are shown in Table 4.

**TABLE 4:** Analysis of variance analysis before and after street-dance training.

Task	Source	*df*	*MS*	*F*	η*^2^_p_*
Go-nogo	Time	1	40.833	1.657	0.028
	Group	1	73.633	2.845	0.047
	Time*Group	1	172.8	7.012*	0.108
ANT	Time	1	208.033	7.532**	0.115
	Group	1	83.333	2.076	0.035
	Time*Group	1	168.033	6.084*	0.085
DCCS	Time	1	208.033	10.55**	0.154
	Group	1	187.5	4.845*	0.077
	Time*Group	1	108.3	5.492*	0.087
Backward digit	Time	1	45.633	13.153**	0.185
span task	Group	1	26.133	5.758*	0.09
	Time*Group	1	16.133	4.65*	0.074

*Significant at α < 0.05; **Significant at α < 0.01.

**FIGURE 1:**
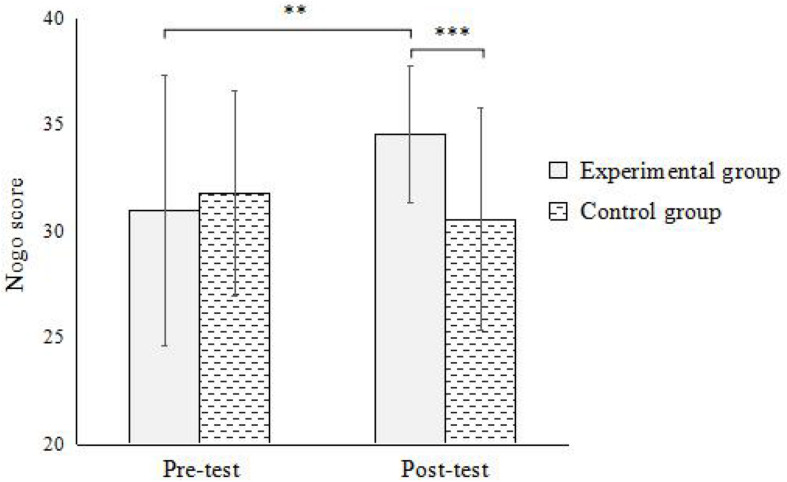
Interaction between time and group in go-nogo task. **Significant at α < 0.01; ***Significant at α < 0.001.

In go-nogo task, the results showed (Figure 1): the interaction between the time points and groups was significant [*F*(1,59) = 7.012, *p* < 0.05, η^2^ = 0.108]. Simple effect analysis found that there was a significant difference between the pre-test and post-test of the experimental group (*p* < 0.01), and there was no significant difference in the pre-test and post-test of the control group (*p* > 0.05). Under the pre-test condition, the difference between the experimental group and the control group was not significant (*p* > 0.05); under the post-test condition, the difference between the experimental group and the control group was significant (*p* < 0.001). This indicates that street-dance training can significantly promote the development of response inhibition in children.

**FIGURE 2:**
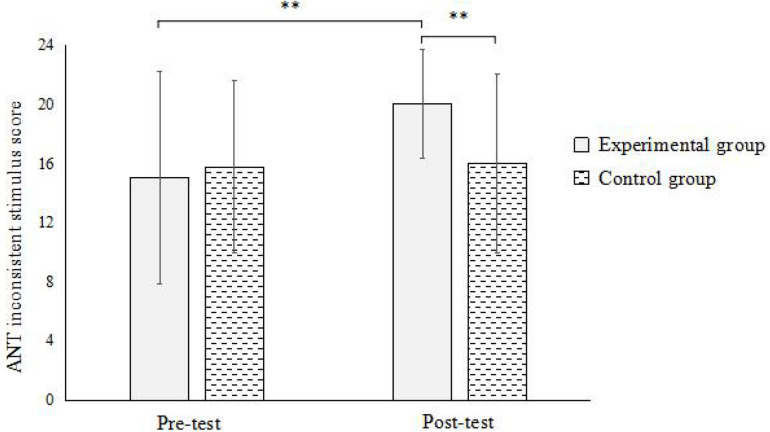
Interaction between time and group in ANT. **Significant at α < 0.01.

In ANT, the results showed (Figure 2): the interaction between the time points and groups was significant [*F*(1,59) = 6.084, *p* < 0.05, η^2^ = 0.095]. Simple effect analysis found that there was a significant difference between the pre-test and post-test of the experimental group (*p* < 0.01), and there was no significant difference in the pre-test and post-test of the control group (*p* > 0.05). Under the pre-test condition, the difference between the experimental group and the control group was not significant (*p* > 0.05); under the post-test condition, the difference between the experimental group and the control group was significant (*p* < 0.01). This indicates that street-dance training can significantly promote the development of executive attention in children.

**FIGURE 3:**
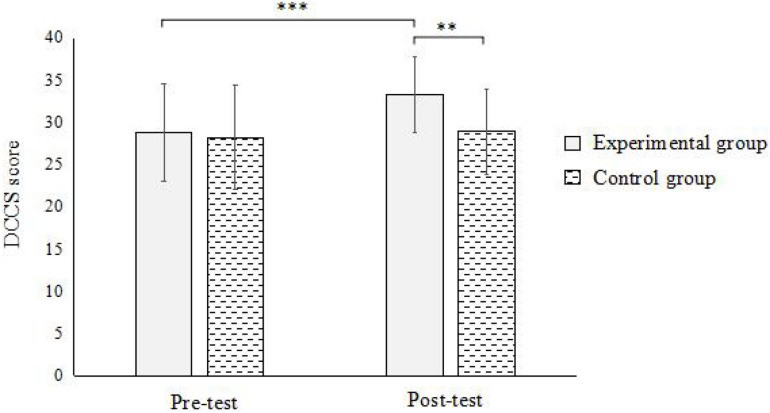
Interaction between time and group in DCCS.

In DCCS, the results showed (Figure 3): the interaction between the time points and groups was significant [*F*(1,59) = 5.492, *p* < 0.05, η^2^ = 0.087]. Simple effect analysis found that there was a significant difference between the pre-test and post-test of the experimental group (*p* < 0.001), and there was no significant difference in the pre-test and post-test of the control group (*p* > 0.05). Under the pre-test condition, the difference between the experimental group and the control group was not significant (*p* > 0.05); under the post-test condition, the difference between the experimental group and the control group was significant (*p* < 0.01). This indicates that street-dance training can significantly promote the development of CF in children.

**FIGURE 4:**
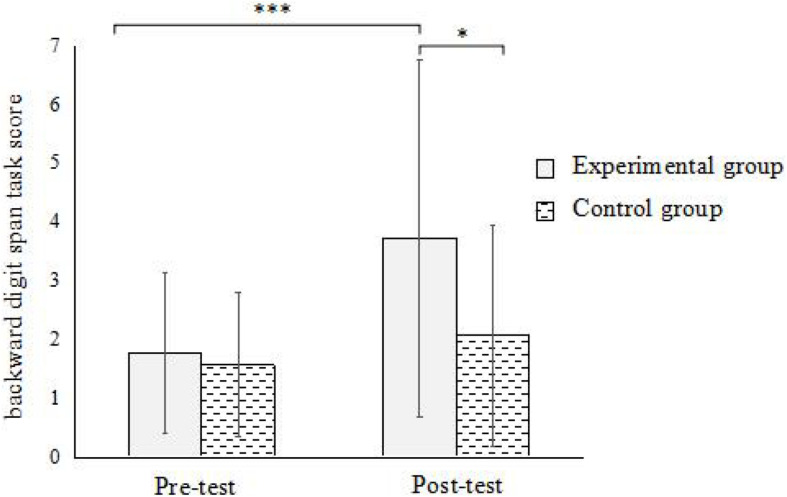
Interaction between time and group in backward digit span task. *Significant at α < 0.05; ***Significant at α < 0.001.

In backward digit span task, the results showed (Figure 4): the interaction between the time points and groups was significant [*F*(1,59) = 4.65, *p* < 0.05, η^2^ = 0.074]. Simple effect analysis found that there was a significant difference between the pre-test and post-test of the experimental group (*p* < 0.001), and there was no significant difference in the pre-test and post-test of the control group (*p* > 0.05). Under the pre-test condition, the difference between the experimental group and the control group was not significant (*p* > 0.05); under the post-test condition, the difference between the experimental group and the control group was significant (*p* < 0.05). This indicates that street-dance training can significantly promote the development of working memory in children.

In order to explore the possible moderating factors of individual differences in training gain, we conducted a set of hierarchical linear regression analysis on the experimental group and the control group. For training gain, we consider that the possible moderating factors are the level of pretest and group.

For each training gain (post-evaluation score T2 minus pre-evaluation score T1), the pre-test level was used as the predictor of the regression analysis in the first step, and then in the second step, the group’s virtual code with scores of 0 and 1 was included regression analysis. Regression analysis showed that the pre-test level was a significant predictor of training gain in the four EF tasks (see Table 5). Moreover, we found that the worse the level of pretest, the higher the training benefit of four EF tasks, which indicates that individuals with poor EF have more benefits in street-dance training. In the second step, the inclusion of group significantly increased the prediction of training gain. This shows that the training gain can be explained by training factors (training group, control group) when individual differences in pretest are controlled.

**TABLE 5:** Summary of hierarchical linear regression analysis for variables predicting gains in tasks.

	Training gains in Go-nogo task		Training gains in ANT task		Training gains in DCCS task		Training gains in backward digit span task	
				
	△*R*^2^	β	△*R*^2^	β	△*R*^2^	β	△*R*^2^	β
Step1	0.588***		0.532***		0.428***		0.089*	
Pre-test		−0.764***		−8.572***		−0.674***		−0.332*
Step2	0.074**		0.072**		0.107**		0.088*	
Group		0.273**		0.269**		0.328**		0.298*
*R*^2^	0.662		0.605		0.536		0.177	
*F*	55.736***		43.586**		32.868***		6.128**	

β is based on the final regression model with all predictors. *Significant at α < 0.05; **Significant at α < 0.01; ***Significant at α < 0.001.

In order to investigate if there were any difference between the gains of experimental group and control group before and after street-dance training, we conducted an independent sample *t*-test. The results are shown in Table 6. The results indicated that the gains from four EF experiments had significant differences between the two groups, which demonstrated great impact of street-dance training on experimental group.

**TABLE 6:** T-test analysis of the gain (T2-T1).

Task	Groups	*N*	*M*	SD	*t*	*df*
Go-nogo	Experimental group	30	3.57	5.606	2.648*	58
	Control group	30	−1.23	8.195		
ANT	Experimental group	30	5	6.56	2.467*	58
	Control group	30	0.27	8.212		
DCCS	Experimental group	30	4.53	5.097	2.344*	58
	Control group	30	0.73	7.273		
Backward digit span task	Experimental group	30	1.97	2.846	2.156*	58
	Control group	30	0.5	2.403		

*Significant at α < 0.05.

## Discussion

In recent years, researchers have continuously explored different ways to promote the development of children’s EF. The flexibility, musicality, and freedom of street dance make it more suitable for the promotion of EF. First of all, in the form of training, street-dance is performed by combining the rhythm rules of music training and the rules of movement in sports training. It can not only solve the boring problems brought by sports training but also solve the participation problem in music training. Therefore, combined with the natural rhythm of music, designing a street-dance training module suitable for children is a relatively comprehensive training method. Second, in terms of the promotion of psychological function, hip-hop can exercise individual perception, body balance, hand-eye coordination, attention, and emotional stability, and then, promote the development of sensory integration. Finally, although many different activities can improve EF, the key factor is that children are willing to spend their time on these activities, so interest and attraction are the main motivation for children to practice repeatedly. Street dance can not only meet the physical needs of children, but also be favored by children because of its variety, low difficulty and easy to learn. Therefore, although different cognitive science training has made progress in promoting individual EF, it is advantageous to use street-dance training to improve children’s EF.

To be close to the daily teaching activities of kindergarten, the street-dance training in this study chose two natural classes as street-dance training group and control group. The number of children in each class was the same, and the street-dance training time was 8 weeks, which was different from the previous related research recruitment subjects. For randomly recruited subjects, their training interest and motivation were low (Corrigall et al., 2013). Second, due to the long training duration, the subjects could have quit halfway. For example, Schellenberg (2004) conducted a 1-year musical training for 144 children, in which 12 subjects quit halfway. The natural classes of the kindergarten selected in this study has no children withdrawing during the 8-week street-dance training. And this street-dance training was more ecological, laying a foundation for the promotion and research of street-dance training.

According to the content structure of EF, this research designed a set of street-dance courses suitable for children. The results showed that 8-week street-dance training can promote the development of children’s EF. Specifically, the scores of WM, IC and CF tasks in the street-dance training group are significantly higher than those in the control group, which is consistent with the hypothesis of our study. But EF is a complex cognitive ability, the sub-components of EF can be separated but correlated (Miller and Best, 2010). Once the correlations with the common EF factor were controlled, shifting and updating tasks captured updating-specific and shifting-specific factors, respectively, whereas inhibition tasks correlated with the common EF virtually perfectly (Miyake and Friedman, 2012). The common EF are assumed to play a role in maintaining goal representations. Therefore, it is possible that the street-dance training would encourage children to keep the teacher’s complete demonstration in mind, coordinate their own action toward the demonstration, and integrate their actions with music, which possibly flourish the ability to maintain goal representations. In the training process, in addition to special training for the three sub-components of EF, common EF factors also play an important role. In order to explain more clearly how the street-dance courses train the internal structure of EF, this research discusses the improvement of the three sub-components separately.

### Influence of Street-Dance Dance Training on Inhibition Control Skill

The results show that street-dance training intervention improves children’s performance on the go-nogo task, which indicates that street-dance training can promote IC. This is consistent with previous studies. Studies have shown that IC ability can be increased by increasing physical activity (Hillman et al., 2009; Chaddock-Heyman et al., 2014; Bidzan-Bluma and Lipowska, 2018; de Greeff et al., 2018). For example, meta-analysis studies have shown that longitudinal physical activity programs have a positive effect on cognitive control (de Greeff et al., 2018).

Response inhibition is an important component of IC. It refers to the ability of individuals to suppress responses that do not meet current needs or inappropriate behaviors (Aron and Poldrack, 2005). A large number of movements are added to our street-dance courses to improve the IC function. There are similar movements between different beats in the street dance curriculum design, and add a lot of reverse action exercises. Children need to learn to distinguish the similarities and differences between the old and new movements, so as to suppress impulse. In addition, IC is the ability to break the habit. Our street-dance course improves the ability of IC by changing the speed of the movement rhythm. By letting the children’s limbs control their speed according to the music rhythm, they can change from dynamic to static in an instant, reflecting to break inertial behavior and improving their IC skill.

### Influence of Street-Dance Dance Training on Cognitive Flexibility Skill

The results showed that the street-dance training intervention improved the participants’ performance on the DCCS task; the street-dance training had a promoting effect on cognitive flexibility skill, which was consistent with the previous research results. Many studies have shown that exercise can improve the CF of individuals.

CF involves the conversion of two different tasks. According to this feature, we designed a street-dance curriculum. First of all, a set of street-dance routine is a combination of several individual movements and basic skills. It is a transformation from one dance movement to another, rather than a single repetition. Therefore, after mastering the basic dance skills and movements, children should also practice how to connect these movements, so that their CF can be better exercised. Second, our street-dance course also adds the cooperation of form transformation. Being familiar with dance movements, children should also remember their own positions. If there are errors in movements and rhythms, they should adjust their movements in time according to the music rhythm. This process also exercises CF.

### Influence of Street-Dance Dance Training on Working Memory Skill

The results show that street-dance training improves children’s performance on the backward digit span task. Street-dance training has a positive effect on children’s WM, which is inconsistent with some previous studies. Dietrich (2006) shows that moderate-intensity exercise intervention does not promote WM, which may be related to the “theory of transient hypofrontality hypofunction.” The prefrontal lobe is responsible for the advanced cognitive function; when moving, the sensory system and the motor action cross each other. When the intensity of the motor action is high, part of the resources responsible for the sensory system will be allocated to the motor system. Thus, some parts of the sensory system will be “closed” early, and the perception will disappear, causing interference to the WM.

WM is the process of storing and processing information. Our street-dance training combines its characteristics. First of all, in terms of motion technology, we designed to repeat the learned motions at different beats. Second, when children learn movements, they must remember the movement name, its characteristics, force parts, and so on, to store this information in the brain, and when they need to use the movement, they can call out the information in time to present the actions. These processes have exercised the WM of children.

Although we will discuss the sub-components of EF separately, the EF is a whole, and the three sub-components are interrelated. Diamond proposed that although IC is the core component of EF, the need to maintain your goals in WM, or what you should and shouldn’t do, is crucial to knowing what to suppress. And the purpose of IC is mainly to adapt to the environment, which requires CF to allow us to get rid of the mental framework, look at problems from many different angles, switch quickly between tasks, or switch routes flexibly when needed (Diamond, 2013).

Therefore, the action design of street-dance training in this study is a process from simple to complex, from single action to action combination, in order to achieve the goal of overall training for EF. In the early training, a single action may point to a certain sub-component, but as the action gradually increases, the action becomes more complex, and children need to remember various rules, so the impact of street-dance training on WM gradually increases. And with the increase of the frequency of movements, the continuous conversion of rules, the coordination of music, the formation of changes, etc., the impact of street-dance training on CF has gradually increased. Therefore, the street-dance training in this study, while cultivating IC, gradually has an effect on WM and CF, thereby affecting the improvement of children’s overall EF.

### The Effect of Individual Differences on Executive Function

In order to explore the influence of individual differences before training on the effect of EF, we conducted a set of hierarchical linear regression analysis on the experimental group and control group. The regression analysis results in the first step show that the worse the pre-test level, the higher the training gains of the four EF tasks, which indicates that individuals with poor EF gain more gains in street-dance training. This is consistent with previous studies (Diamond and Lee, 2011; Diamond and Ling, 2016). Diamond suggested that those with the worst EF always benefit the most from training to improve EF, children with low income, low WM span, and ADHD, as well as boys (who tend to have worse I than girls, Moffitt et al., 2011) generally show better EF improvement during training (Diamond and Lee, 2011). Moreover, since those who started late in EF tend to make greater progress in EF intervention, EF training may reduce differences in EF related to academic achievement and/or health (at least to some extent giving the laggards a chance to catch up) (Diamond and Ling, 2016). This gives us great hope that those children who do not perform well in EF are likely to be temporary, and when we provide them with proper street-dance training, they will make progress, which will greatly reduce their later achievement gap with other children.

In the second step, the inclusion of group significantly increased the prediction of training gain. This shows that the training gain can be explained by training factors when individual differences in pretest are controlled. And we further explored whether there is a difference between the gains of experimental group and control group before and after street-dance training. The results show that street-dance training has a greater impact on the experimental group. In short, after controlling for the pre-test factors, we found that street-dance training does promote the development of children’s EF.

### Research Prospects and Limitations

First, our study only explored the impact of street-dance training on the EF of preschool children, and the age group of participants was the same. Future research will further explore the effect of street-dance training on children of different ages. Second, our study used blank control rather than active control. It is unclear whether other non-training factors will affect the training results. Future research may try to use active control groups, such as experimental group (street-dance training)/control group (relaxation training), to further investigate the impact of street-dance training on children’s EF after excluding other non-specific factors. Third, our study lacked the measurement of the delay effect. In the following research, we will further explore the delay effect and explore the maintenance effect of the training program. Fourth, since the common EF factor also plays an important role in street-dance training, the promotion of the various sub-components of EF by street dance training discussed in this study only represents a possibility. Therefore, in order to demonstrate the specific impact of street-dance training on each sub-component of EF, more research needs to be done in the future to ensure that street-dance training for each sub-component only improves that function. Finally, future research can further expand the study of dependent variables and explore the impact of street-dance training on other cognitive characteristics of children.

## Conclusion

In this study, we used a standard experimental paradigm system to comprehensively examine the impact of street-dance training on the EF of preschool children. It was found that street-dance training can improve children’s WM, IC, and CF, which provides new methods for early training of children’s EF, and has important theoretical guidance value and practical significance.

## Data Availability Statement

The raw data supporting the conclusions of this article will be made available by the authors, without undue reservation.

## Ethics Statement

The studies involving human participants were reviewed and approved by Liaoning Normal University. Written informed consent to participate in this study was provided by the participants’ legal guardian/next of kin.

## Author Contributions

YS and LF determined the research ideas and methods. QZ carried out experimental operation, training, data analysis and writing articles. YH and GL helped with the training. All authors contributed to the article and approved the submitted version.

## Conflict of Interest

The authors declare that the research was conducted in the absence of any commercial or financial relationships that could be construed as a potential conflict of interest.
